# Early Caffeine Exposure Causes Metabolic and Hormonal Changes Differently According to the Window of Exposure (Gestation or Lactation), Sex, and Age in a Rat Model

**DOI:** 10.3390/nu17172763

**Published:** 2025-08-26

**Authors:** Luana Lopes de Souza, Rosiane Aparecida Miranda, Iala Milene Bertasso, Beatriz Souza da Silva, Mayara da Silva Almeida, Reinaldo Röpke-Junior, Beatriz Ribeiro de Oliveira, Leandro Miranda-Alves, Egberto Gaspar Moura, Patricia Cristina Lisboa

**Affiliations:** 1 Laboratory of Endocrine Physiology, Institute of Biology Roberto Alcantara Gomes, Universidade do Estado do Rio de Janeiro, Rio de Janeiro 28905-320, RJ, Brazil; roapmiranda@yahoo.com.br (R.A.M.); imbertasso@gmail.com (I.M.B.); biabss1@gmail.com (B.S.d.S.); almeidamayy21@hotmail.com (M.d.S.A.); egmoura3@gmail.com (E.G.M.); pclisboa.uerj@gmail.com (P.C.L.); 2 Laboratory of Experimental Endocrinology-LEEx, Institute of Biomedical Sciences, Federal University of Rio de Janeiro, Rio de Janeiro 21941-902, RJ, Brazil; reiropke@hotmail.com (R.R.-J.); beaoliveira@ufrj.br (B.R.d.O.); leandro.alves@icb.ufrj.br (L.M.-A.)

**Keywords:** maternal caffeine, lactation, gestation, DOHaD, obesity development, fructose overload

## Abstract

**Background/Objectives:** Many women report restrictions on caffeine intake during gestation, but some of these restrictions are withdrawn during the lactation period. Given that both periods have elevated epigenetic plasticity, our aim was to compare the effects of caffeine exposure during each isolated period on offspring metabolism and susceptibility to obesity in response to metabolic overload. **Methods:** Pregnant Wistar rats received orogastric caffeine (CAF) (25 mg/kg/day) or vehicle during gestation (CAF G) or lactation (CAF L) periods. We evaluated the body mass, adiposity, hormone levels, and food behavior of offspring of both sexes at different ages. Adult animals were subjected to metabolic overload, with fructose solution (10%) offered for ten days. **Results/Discussion:** CAF G and CAF L dams presented lower T3 levels (−70 and −52%) because of reduced TSH activity in the thyroid gland (−28 and −29%), despite unchanged gland morphology. At weaning, CAF G and CAF L males presented lower T3 levels (−75 and −80%), as did CAF L females (−85%). At puberty, females in the CAF L group showed glucose intolerance. In adulthood, CAF G males exhibited a greater preference for palatable food. In addition, CAF G and CAF L males showed increased feed efficiency, suggesting a greater susceptibility to obesity development. To test this susceptibility, the animals were subjected to fructose overload. Indeed, we observed that despite the absence of a fructose effect in the control group, male CAF G and female CAF L animals showed greater adiposity in response to fructose overload (+43% and +37%, respectively). **Conclusions:** Caffeine exposure during lactation increases the risk of obesity development among female offspring. However, for male offspring, gestation seems more critical.

## 1. Introduction

Early life is a critical period of development in which intense epigenetic plasticity exists, susceptible to environmental factors [1]. Therefore, inadequate maternal diet or factors related to maternal diet can modify epigenetic mechanisms, promoting adaptive changes in offspring with short- and long-term repercussions [2]. This adaptive response could match or mismatch the future environment, explaining individual susceptibility to health or diseases, as described by the Developmental Origins of Health and Disease (DOHaD) concept [2,3]. This concept helps explain the increased individual risk of metabolic dysfunction in response to a metabolic insult, such as a high-fat diet (HFD) [4] or fructose overload [5,6,7].

The intrauterine period is a classical critical window of development. However, some physiological systems are also in development after birth; during neonatal life, lactation is also a critical window of development and is susceptible to DOHaD [8]. Some metabolic tissues, such as adipose tissue and pancreas, are not only in development during gestation but also during the lactation period, resulting in differences in ontogenetic plasticity during the perinatal period [9]. A maternal cafeteria diet during the lactation period promoted greater adiposity in male offspring at weaning than in offspring exposed during gestation [10]. On the other hand, maternal undernutrition promoted higher adiposity and hyperleptinemia in adult offspring only when it was offered during pregnancy but not during lactation [11]. An insult during the gestation or lactation period could subsequently differentially impact offspring metabolism throughout life.

Our research group has demonstrated the important effects of the lactation period on offspring metabolism and endocrine physiology throughout life [8]. This is a concern since there are nutritional recommendations specific to pregnant women, but few recommendations specific to lactating women exists. In addition, some women follow the restrictions during pregnancy, but the restrictions are withdrawn after baby birth, exposing the baby precociously to insults. Among these gestational restrictions is maternal caffeine intake. This caffeine intake during the perinatal period is a concern, as there is an increase in the maternal half-life of caffeine during the gestation of humans and rodents, increasing the exposure of the fetus, which is unable to metabolize this substance [12]. In addition, during the lactation period of humans and rodents, maternal caffeine can pass to the baby through breastmilk, exposing the baby, which exhibits immaturity in caffeine metabolism [13].

Almost 70% of American women maintain their caffeine intake during pregnancy [14], with a reduction in median intake to 160–190 mg/day [14,15]. However, 48.1% of breastfeeding women exceed a daily intake of 200 mg [16]. Purkiewicz et al. (2022) reported that almost 70% of nursing women consumed coffee more than twice a day, reaching a range of caffeine intake from 200 to as much as 400 mg/day [17]. Indeed, the milk of these lactating women contained increased levels of caffeine metabolites [17].

There is epidemiological and experimental evidence that excess maternal caffeine during gestation is correlated with higher risk of miscarriage [18] and low birth weight [19,20], in addition to other physiological and behavioral changes in offspring [4,21]. On the other hand, caffeine excess during lactation is associated with the occurrence of infant colic, atopic dermatitis, hyperactivity, hyperglycemia, and tachycardia [22]. Therefore, the World Health Organization (WHO) recommends limiting maternal caffeine intake to 300 mg per day throughout the gestation period [15,23,24].

Caffeine is a methylxanthine present in coffee, tea, chocolate, and some soft drinks [12]. Caffeine and other methylxanthines are lipophilic substances that can pass through the placental and mammary gland barriers [25,26], and are found in amniotic liquid, in the fetal compartment [20,27], and in breast milk from caffeine users. In rodents, caffeine levels in breast milk are proportional to caffeine levels in the plasma and brains of pups [28]. This is a concern because the baby only develops the ability to metabolize caffeine after six months of age [13]. Therefore, even a low maternal intake of caffeine, lower than the WHO limits, may not be safe for offspring [12,29].

Previously, we demonstrated that a low intake of caffeine during the continuous period of gestation and lactation promoted hormonal, metabolic, and behavioral changes in offspring of both sexes [29]. Early exposure to caffeine in animal models resulted in important changes in thyroid function, promoting early hypothyroidism and hyperthyroidism in adulthood [29]. In addition, adult male offspring exhibited greater locomotor activity and a stronger preference for a palatable diet, whereas female offspring exhibited lower insulin levels and hyperandrogenism [29]. This phenotype reflects the development of many adaptive mechanisms that can depend on the period of caffeine exposure.

Therefore, despite the evident harmful effect on offspring health of low caffeine exposure during the continuous perinatal period, the impacts of this low dose during the isolated periods of gestation or lactation are not clear. Our hypothesis is that different effects of caffeine, according to the perinatal period, impact glucose homeostasis and adiposity, since the endocrine pancreas and adipose tissue are organs in development during the lactation period in both humans and rodents [30]. In addition, we believe that these adaptive mechanisms can increase individuals’ sensitivity to metabolic overload, such as that promoted by nutritional habits, such as fructose overload. Fructose seems to be an important insult in this model, because it quickly promotes metabolic disturbance, increasing insulin resistance and adiposity [31], and because of the elevated intake of soft drinks rich in fructose and caffeine [31]. The aim of this study was to compare the morphometrical, metabolic, and hormonal profiles of offspring exposed to caffeine during the gestation or lactation period and corresponding responses to fructose overload in adulthood.

## 2. Materials and Methods

### 2.1. Experimental Design

The experimental design was approved by the Ethics Committee on Animal Care of the Biology Institute of the State University of Rio de Janeiro (CEUA/026/2019; approved in November, 2019) as required by the Brazilian Law no. 25 11.794/2008 [32], in accordance with the ARRIVE guidelines [33]. The animals were kept in a room with controlled temperature (22 ± 2 °C), humidity, and light (light/dark cycles: lights on at 7 a.m. and lights off at 7 p.m.). The animals had free access to water and chow (Nuvilab, São Paulo, Brazil).

Mating was performed using two female Wistar rats with one male rat (10 weeks of age), and the presence of sperm in the vaginal fluid (gestational day 0) was checked daily. On gestational day 3, pregnant rats were randomly divided into three groups: control (C, *n* = 6), caffeine gestation (CAF G, *n* = 5), and caffeine lactation (CAF L, *n* = 6). Pregnant rats were given daily to vehicle (water) or caffeine solution (1,3,7-trimethylxanthine; Proquimios, Rio de Janeiro, Brazil) daily by oral gavage. The CAF G group received caffeine solution from gestational day 3 until birth, while the CAF L group received caffeine solution from birth until the 21st postnatal day (the end of the lactation period). CAF groups received a dosage of 25 mg/kg/day of caffeine, which is equivalent to 250 mg/day of caffeine intake by pregnant women [34]. This dosage is less than 300 mg/day, a safe dosage during pregnancy according to the WHO [24], which corresponds to approximately 2 cups of coffee per day [35].

After delivery, only litters of 9 to 12 pups remained in the experiment. At birth (postnatal day 0—PND0), the litters were adjusted to 8 pups per dam, with 4 males and 4 females. At weaning (PND21), the dams and one male and one female from each litter were euthanized after overnight fasting. The remaining offspring were evaluated throughout life. Therefore, we used a total of 136 animals, including female and male offspring.

At PND 170, offspring from the control and caffeine groups were divided into two groups: one that received drinking water and another that received a fructose solution (10%) until euthanasia. D-fructose was dissolved in the drinking water (Sigma Aldrich, MO, USA), and the intake of the solution was measured every two days. The fructose solution concentration is close to the fructose concentration of some soft drinks [36]. Chow intake was measured weekly. At 180 days of age, the animals were euthanized by decapitation after being fasted overnight, in the absence of chow or fructose solution. The offspring had free access to standard rodent chow during the experiment (Nuvilab). With respect to female euthanasia, all females were killed during the diestrus phase to reduce the interference of the estrous cycle.

Blood samples were obtained from the trunk in heparinized tubes, and, after centrifugation (1200× *g* for 15 min at 4 °C), the plasma was kept frozen at −20 °C. The thyroid gland (dams and offspring), adrenal gland, brown adipose tissue, and liver were dissected and kept for subsequent analyses.

The fat mass of the dams was evaluated at 20 days after delivery, and of the offspring at weaning (PND21), by nuclear magnetic resonance (NMR). Animals were immobilized in a plastic cylinder with a tight-fitting plunger and were scanned (for 2 min) using whole-body composition analyzer NMR equipment (Bruker Minispec LF90 TD-NMR, Rheinstetten, Germany). The data are expressed as the percentage (%) of fat mass.

From PND75 until PND150, we evaluated food intake, as the sum of all chow intake by the animal and body mass gain, as the difference in body mass at PND150 and PND75. Using these data, we calculated the feed efficiency ratio of the animals as the ratio of body mass gain (g) to the accumulated food intake (g) during this period [37].

### 2.2. Breast Milk Composition

Two hours after maternal separation of the litters, the dams were subjected to intraperitoneal oxytocin (5 UI/animal i.p.) and anesthesia (thiopental 30 mg/kg BW i.p.), followed by manual milk collection. As previously described [38], the content of lactose in the breastmilk was measured by a colorimetric assay based on picric acid reduction [39]; protein content was measured by a colorimetric assay [40]; and triglyceride and cholesterol contents were measured by a colorimetric commercial kit (Bioclin, Belo Horizonte, Brazil). In addition, we measured insulin (#EZRMI-13K) and leptin (#EZRL-83K) levels in the milk using a commercial ELISA kit according to the manufacturer’s recommendations (Merck Millipore, Darmstadt, Germany).

### 2.3. Biochemical and Hormonal Measurements

Plasma triglyceride and cholesterol levels were measured by a colorimetric commercial kit (Bioclin). Plasma insulin (sensitivity: 0.2 ng/mL; #EZRMI-13K; Merck Millipore), leptin (sensitivity: 0.04 ng/mL; #EZRL-83K; Merck Millipore), total T3 (sensitivity: 0.094 ng/mL; #ER1720; Fine test, Wuhan, China), free T4 (sensitivity: 0.938 pg/mL; #ER0962; Fine test), and TSH (sensitivity: 0.75 ng/mL; #ER1411; Fine test) levels were measured by commercial specific rat enzyme-linked immunosorbent assay (ELISA) kits in accordance with manufacturer’s recommendations. Plasma corticosterone levels were measured by a MILLIPLEX^®^ MAP Rat Stress Hormone Magnetic Bead Panel (sensitivity: 1796 pg/mL; #RSHMAG-69K; Merck Millipore).

### 2.4. Oral Glucose Tolerance Test (OGTT)

At puberty (PND45), we performed an OGTT and measured blood glucose levels from tail incision in the animals. Blood glucose was measured using a glucometer after overnight fasting (time 0) and after oral glucose administration (2 g/kg of body mass). We measured glycemia at 15, 30, 60, and 90 min after glucose administration to perform a curve analysis.

### 2.5. Palatable Food Preference Test

In adulthood (PND100), a palatable food preference test using a high-fat diet (HFD) was carried out as described previously [41,42]. Briefly, we offered an equal amount of a standard chow (STD) and an HFD (42% of calories as fat, with lard being the lipid source) (Prag soluções, Jaú, São Paulo, Brazil) (Table 1) and measured food intake during a 12 h period (lights off period) [38]. The relative preference for HFD was estimated by the proportion of HFD intake in relation to the total food intake (standard chow intake plus HFD intake).

At PND110, we also performed a sucrose preference test, simultaneously offering one bottle of water and one bottle of sucrose solution (4%) in each cage. We quantified the volumes of water or sucrose solution intake over 24 h [43]. The relative preference for sucrose was considered the percentage of sucrose solution intake in relation to the total fluid intake during the 24 h period.

### 2.6. Thyroid Morphological Analyses

As previously described [44], one lobe of the thyroid was fixed in 4% paraformaldehyde for 48 h. The samples then underwent a series of steps for dehydration, clarification, and paraffin embedding. Sections 5 µm thick were prepared using a microtome (Leica RM2125 RTS, Leica Biosystems, Nussloch, Germany). The histological sections were stained with hematoxylin and eosin (HE) for morphology analysis. Images were captured at 200× magnification using a microscope (Olympus BX60, Olympus, Tokyo, Japan) equipped with a camera (Retiga 2000R, QImaging, Surrey, BC, Canada). Five randomly selected fields from each thyroid section of each animal were analyzed using ImageJ 1.47 software (https://imagej.net/ij/, 22 July 2025). The parameters measured were the thyroid follicle area, colloid area, epithelial area, and epithelial height; the epithelial/colloid ratio was subsequently calculated for each animal.

### 2.7. Thyroid mRNA Expression

Total RNA from one lobe of each thyroid sample was extracted using the SV Total RNA Isolation System (Promega, Madison, WI, USA) according to the manufacturer’s protocol. cDNA synthesis was performed using a High-Capacity cDNA Reverse Transcription Kit (Applied Biosystems, Foster City, CA, USA) with 1 or 0.5 μg of total RNA (adults or weaned animals, respectively) in a single reaction. mRNA expression was evaluated by qPCR using GoTaq qPCR Master Mix (Promega, WI, USA) and a 7500 Real-Time PCR System (Applied Biosystems, CA, USA). The PCR program was as follows: denaturation for 2 min at 95 °C, 40 cycles of 15 s at 95 °C, and 60 s at 60 °C, following the melting program. The primer sequences are shown in Table 2 for the genes encoding thyroid-stimulating hormone receptors (*Tshr*) and reference genes (*Hprt* and *Rplp0*). The efficiency range accepted for each assay was 90–105%. qPCR quality and genomic DNA contamination was checked using intron-spanning primers, reverse transcriptase-negative controls, and melting curve analysis obtained from each reaction. Relative mRNA expression was calculated using the standard curve method, and the expression level was normalized to the geometric mean of the reference gene values (*Hprt* and *Rplp0*). The results are expressed relative to the values of the control group.

### 2.8. Statistical Analysis

First, we evaluated the normality of the distributions of the variables using the Kolmogorov–Smirnov one-sample test (K–S). Next, to compare the experimental groups for each sex, we used a one-way ANOVA followed by the Dunnet post hoc test. For the analysis of fructose overload, we compared the water and fructose groups within each maternal condition using Student’s t test. Multiple t tests were corrected by the Holm–Sidak method. Statistical analyses were performed with GraphPad Prism 6.0 software (GraphPad Software Inc., San Diego, CA, USA), and *p* < 0.05 was considered to indicate statistical significance. The data are shown as the mean and standard error pf the mean (SEM).

## 3. Results

**Figure 1:**
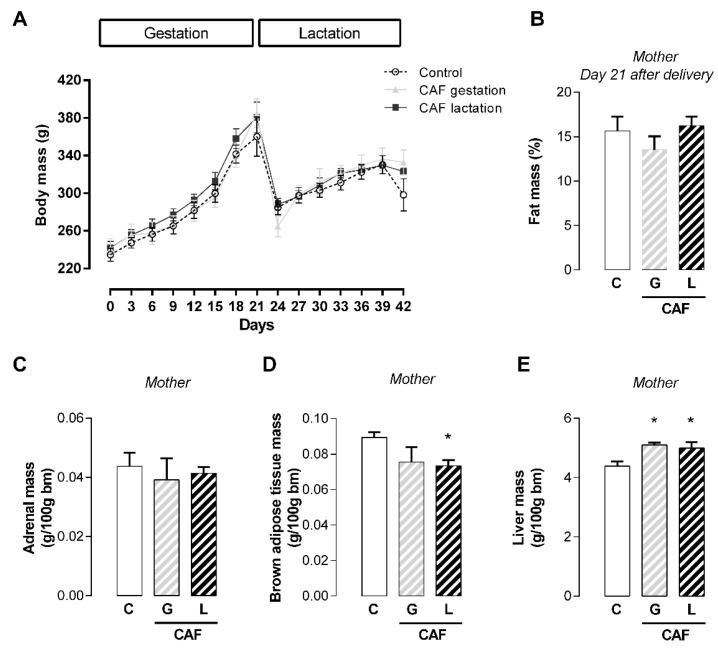
Effects of caffeine intake during gestation (G) or lactation (L) period on maternal morphometric parameters. (**A**) Body mass; (**B**) percentual of fatty mass; (**C**) adrenal mass; (**D**) brown adipose tissue mass; (**E**) liver mass; data are expressed as means ± SEM; *n* = 5–6. Statistical analysis was performed using one-way ANOVA followed by Dunnet’s post hoc test (* *p* < 0.05).

**Figure 2:**
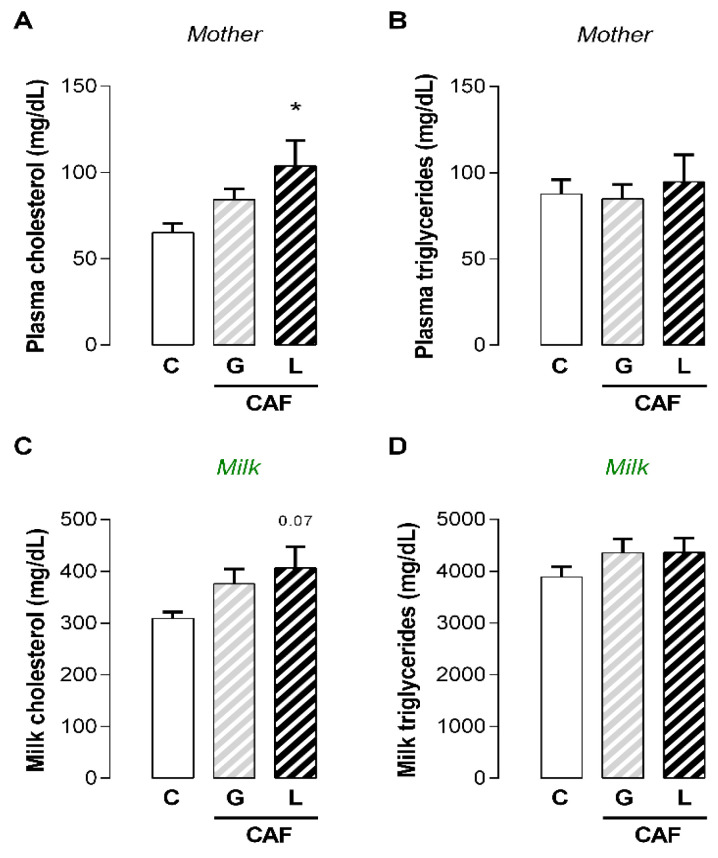
Effects of caffeine intake during gestation (G) or lactation (L) period on maternal plasma lipids and breastmilk composition. (**A**) Plasma cholesterol; (**B**) plasma triglycerides; (**C**) milk cholesterol; (**D**) milk triglycerides; data are expressed as means ± SEM; *n* = 5–6. Statistical analysis was performed using one-way ANOVA followed by Dunnet’s post hoc test (* *p* < 0.05).

**Figure 3:**
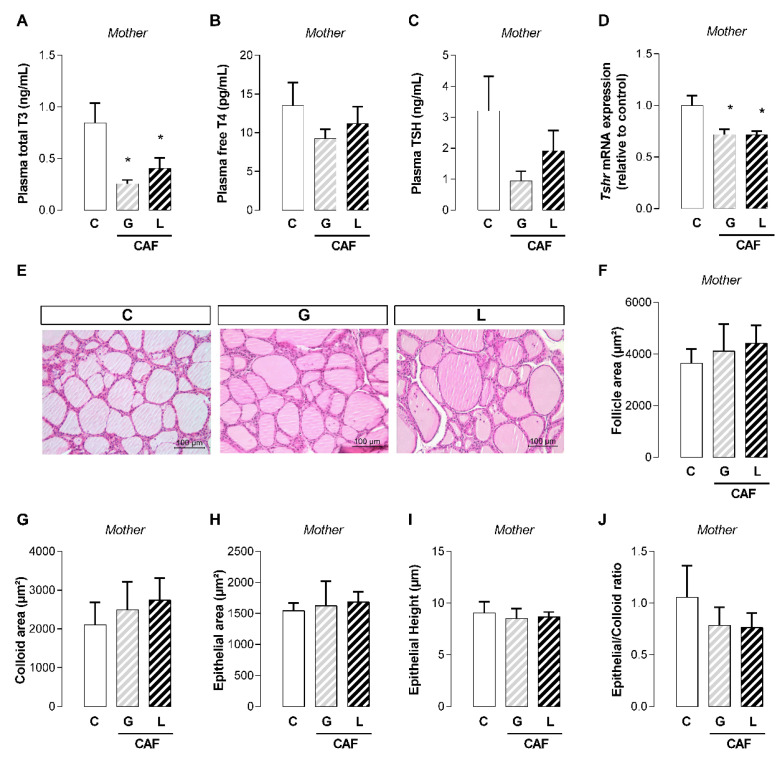
Effects of caffeine intake during gestation (G) or lactation (L) period on maternal thyroid function. (**A**) Total T3; (**B**) free T4; (**C**) TSH; (**D**) *Tshr* mRNA; (**E**–**J**) thyroid gland morphology; data are expressed as means ± SEM; *n* = 5–6. Statistical analysis was performed using one-way ANOVA followed by Dunnet’s post hoc test (* *p* < 0.05).

Maternal caffeine intake during gestation or lactation periods did not affect maternal body mass gain (Figure 1A) or fat mass at the end of lactation (Figure 1B). Although caffeine did not affect maternal adrenal weight (Figure 1C), exposure to caffeine during lactation (L) promoted lower brown adipose tissue mass (−18%; *p* < 0.05) (Figure 1D), whereas exposure to caffeine during gestation (G) and lactation (L) periods promoted greater liver mass in the dams exposed to caffeine (+16 and +14%, respectively; *p* < 0.05) (Figure 1E). At the end of the lactation period, CAF L dams presented higher plasma cholesterol levels (+60%; *p* < 0.05) (Figure 2A), with a trend toward higher cholesterol content in the breastmilk (*p* = 0.06) (Figure 2C) (Table 3). With respect to hormonal changes, caffeine intake did not affect maternal plasma leptin, insulin, or corticosterone levels (Table 4). However, regardless of the period of caffeine intake, caffeine promoted lower maternal plasma T3 levels (−70 and −52%, respectively; *p* < 0.05) (Figure 3A) but did not affect plasma T4 levels (Figure 3B). Although the plasma TSH level was unchanged (Figure 3C), the *Tshr* mRNA expression was lower in the CAF dams than in the control dams (−28 and −29%, respectively; *p* < 0.05), regardless of the duration of caffeine intake (Figure 3D). However, these changes did not significantly modify maternal thyroid morphology (Figure 3E–J).

**Figure 4:**
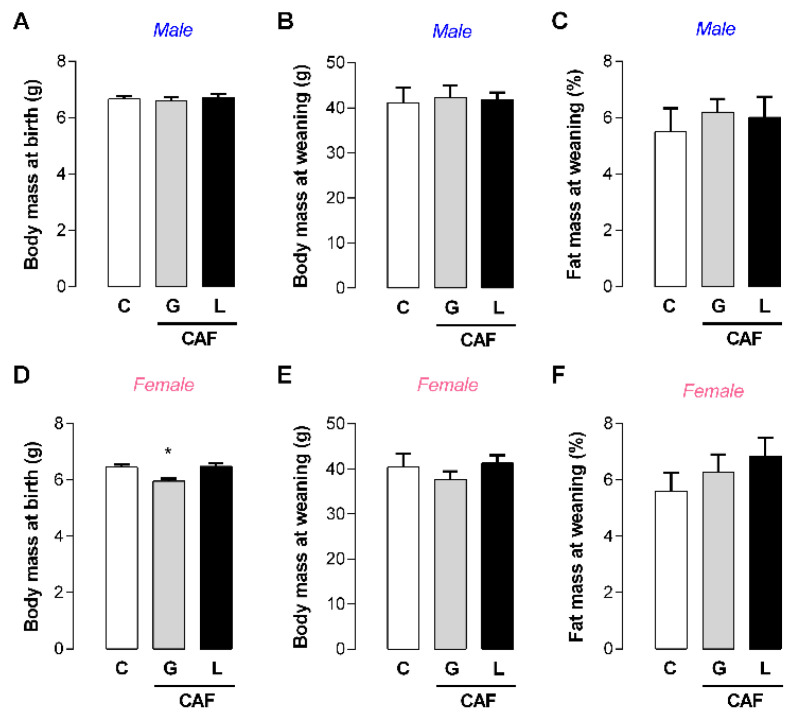
Effects of caffeine exposure during gestation (G) or lactation (L) period on body mass and adiposity in male and female offspring. (**A**,**D**) Body mass at birth; (**B**,**E**) body mass at weaning; (**C**,**F**) fat mass at weaning; data are expressed as means ± SEM; *n* = 5–6. Statistical analysis was performed using one-way ANOVA followed by Dunnet’s post hoc test (* *p* < 0.05).

With respect to the offspring, only the female offspring from the dams exposed to caffeine during gestation presented lower body masses at birth (−8%; *p* < 0.05) (Figure 4D), with no significant changes at weaning (Figure 4E).

**Figure 5:**
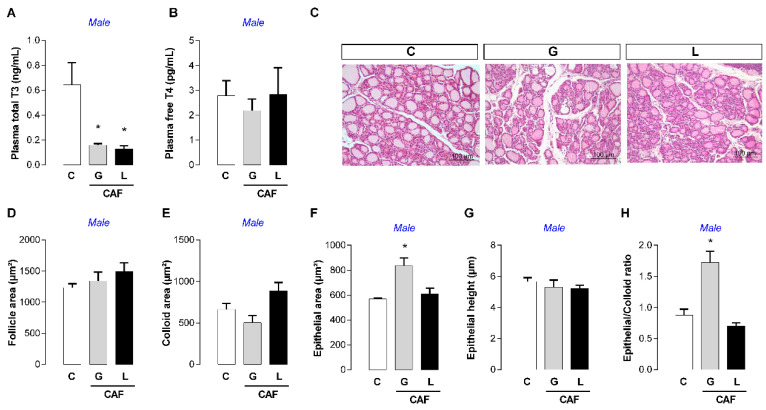
Effects of caffeine exposure during gestation (G) or lactation (L) period on thyroid function of weaned male offspring. (**A**) Total T3; (**B**) free T4; (**C**–**H**) thyroid gland morphology; data are expressed as means ± SEM; *n* = 5–6. Statistical analysis was performed using one-way ANOVA followed by Dunnet’s post hoc test (* *p* < 0.05).

**Figure 6:**
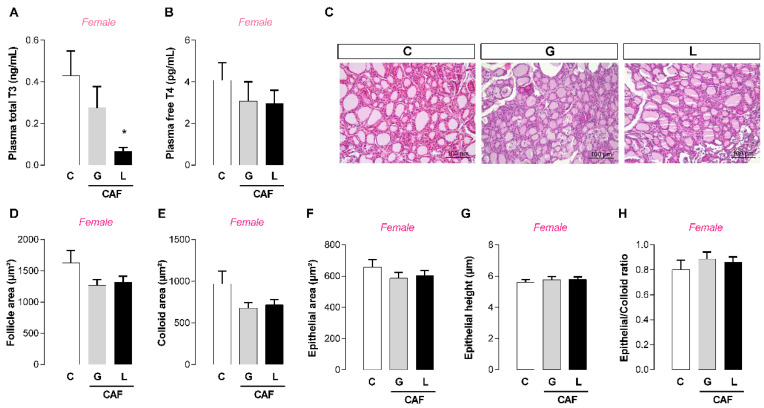
Effects of caffeine exposure during gestation (G) or lactation (L) period on thyroid function in weaned female offspring. (**A**) Total T3; (**B**) free T4; (**C**–**H**) thyroid gland morphology; data are expressed as means ± SEM; *n* = 5–6. Statistical analysis was performed using one-way ANOVA followed by Dunnet’s post hoc test (* *p* < 0.05).

**Figure 7:**
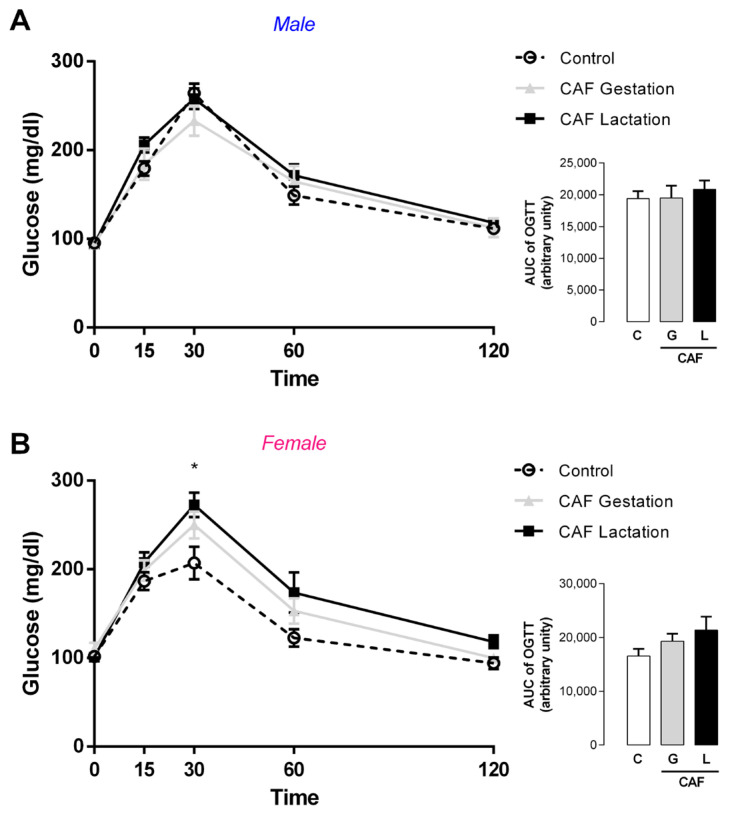
Effects of caffeine exposure during gestation (G) or lactation (L) period on glucose tolerance at puberty in male and female offspring. (**A**) Male OGTT curve and AUC; (**B**) female OGTT curve and AUC; OGTT—oral glucose tolerance test; AUC—area under curve; data are expressed as means ± SEM; *n* = 5–6. Statistical analysis was performed using one-way ANOVA followed by Dunnet’s post hoc test (* *p* < 0.05).

At weaning, male offspring had lower plasma T3 levels (−75 and −80%, respectively; *p* < 0.05) (Figure 5A), and no significant changes in T4 or thyroid morphology, except for a higher epithelial/colloid ratio (+96%; *p* < 0.05) (Figure 5F). Only CAF L female weaned offspring had lower plasma T3 levels (−85%; *p* < 0.05) (Figure 6A), and no significant changes in T4 or thyroid morphology were detected (Figure 6). Both offspring exhibited unchanged plasma leptin, insulin, and corticosterone levels (Table 4). At puberty (PND45), we performed an oral glucose tolerance test (OGTT). After 30 min of test, only the CAF L female offspring showed higher glycemia levels (+32%; *p* < 0.05) (Figure 7B), suggesting glucose intolerance, despite the lack of significant changes in the area under the curve.

**Figure 8:**
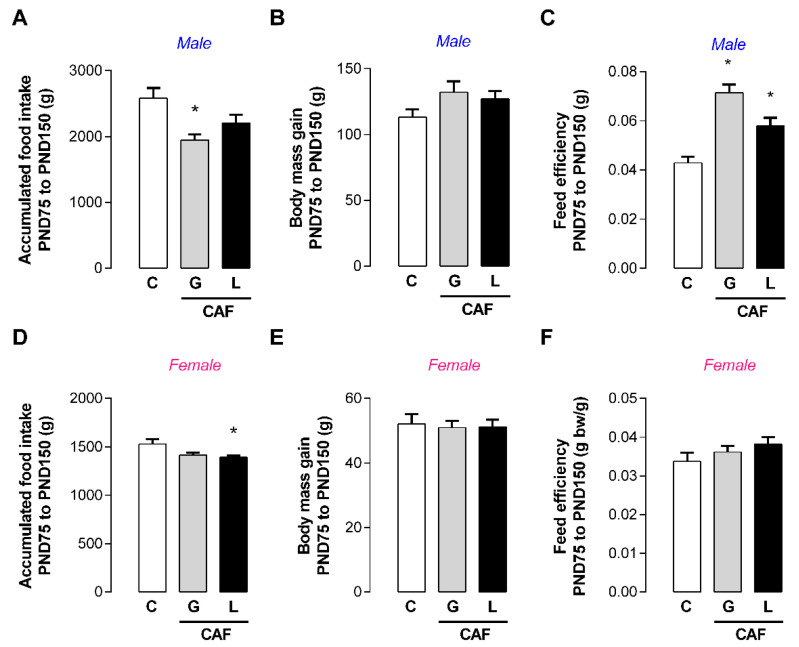
Effects of caffeine exposure during gestation (G) or lactation (L) period on food intake and body mass gain in adult male and female offspring. (**A**,**C**) Accumulated food intake; (**B**,**D**) body mass gain; (**E**,**F**) feed efficiency ratio; PND—postnatal day; data are expressed as means ± SEM; *n* = 5–6. Statistical analysis was performed using one-way ANOVA followed by Dunnet’s post hoc test (* *p* < 0.05).

**Figure 9:**
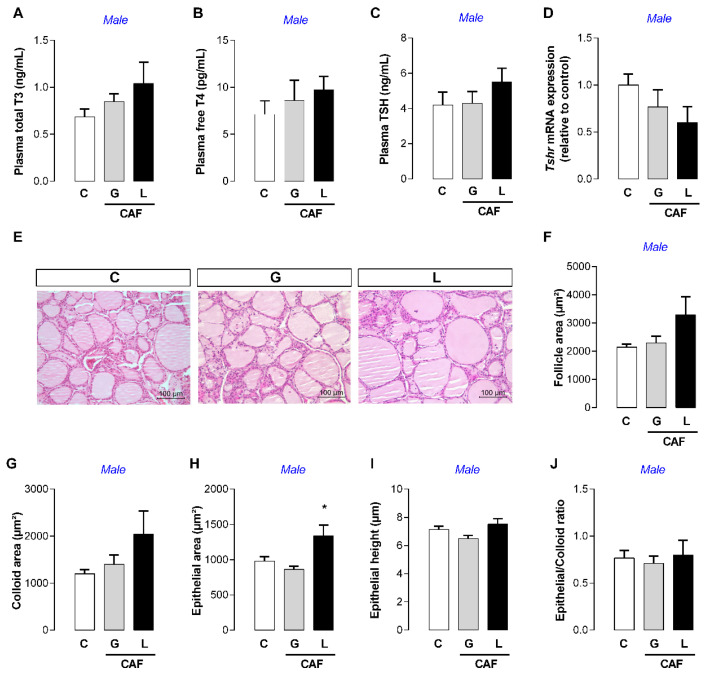
Effects of caffeine exposure during gestation (G) or lactation (L) period on thyroid function in adult male offspring. (**A**) Total T3; (**B**) free T4; (**C**) TSH; (**D**) *Tshr* mRNA; (**E**–**J**) thyroid gland morphology; data are expressed as means ± SEM; *n* = 5–6. Statistical analysis was performed using one-way ANOVA followed by Dunnet’s post hoc test (* *p* < 0.05).

**Figure 10:**
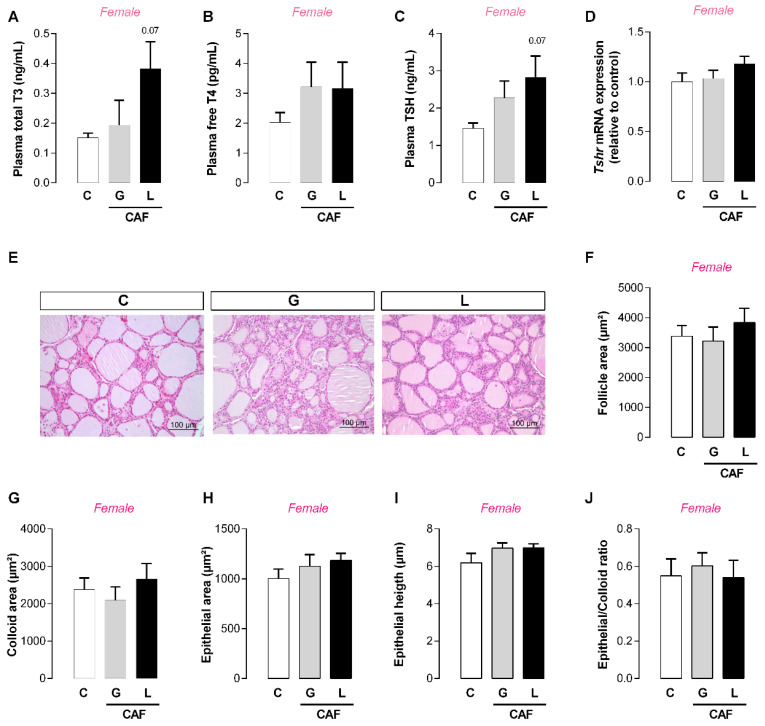
Effects caffeine exposure during gestation (G) or lactation (L) period on thyroid function of adult female offspring. (**A**) Total T3; (**B**) free T4; (**C**) TSH; (**D**) *Tshr* mRNA; (**E**–**J**) thyroid gland morphology; data are expressed as means ± SEM; *n* = 5–6. Statistical analysis was performed using one-way ANOVA followed by Dunnet’s post hoc test.

In adulthood, CAF G male offspring and CAF L female offspring had lower food intake (−25 and −9%, respectively; *p* < 0.05) (Figure 8A,C), but the body mass did not significantly change (Figure 8D,E), suggesting energy imbalance. Indeed, CAF G males had a higher feed efficiency ratio (+67%; *p* < 0.05) (Figure 8C). We evaluated thyroid function and observed a greater epithelial area of the thyroid (+36%; *p* < 0.05) (Figure 9J) in CAF L males, without changes in thyroid hormone levels (Figure 9A,B) or TSH levels (Figure 9C). In female offspring, despite the absence of changes in thyroid morphology, we observed a trend toward higher T3 and TSH levels in the CAF L group (*p* = 0.07) (Figure 10A). With respect to other hormones, the plasma leptin, insulin, and corticosterone levels of offspring of both sexes were unchanged (Table 5).

**Figure 11:**
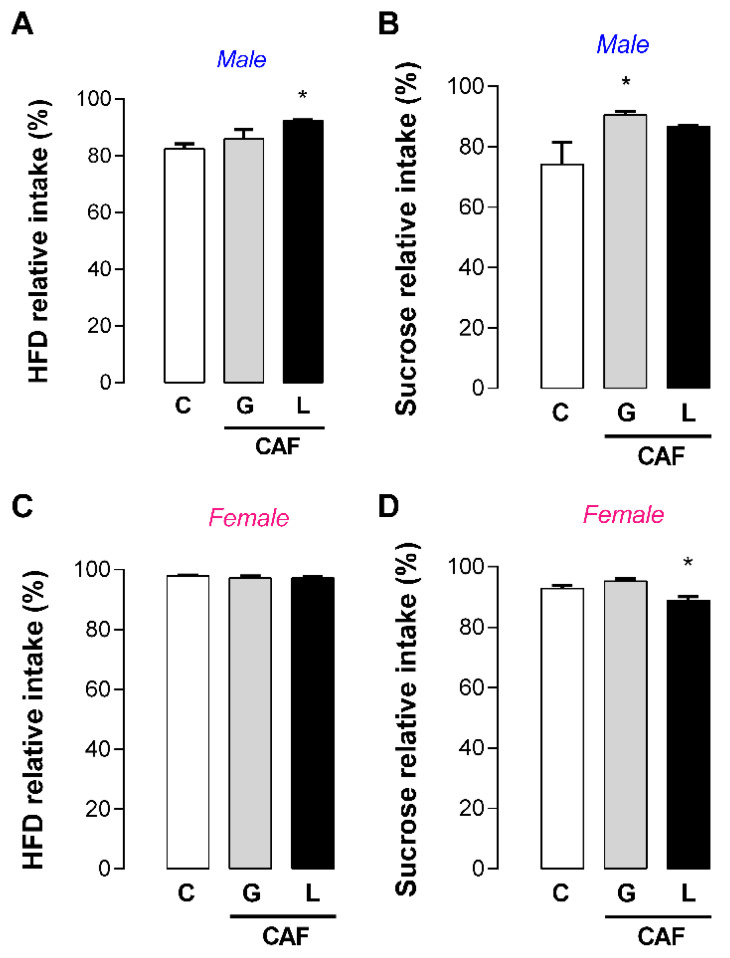
Effects of caffeine exposure during gestation (G) or lactation (L) period on palatable food preference test in adult male and female offspring. (**A**,**C**) Preference for HFD test; (**B**,**D**) preference for sucrose test; data are expressed as means ± SEM; *n* = 5–6. Statistical analysis was performed using one-way ANOVA followed by Dunnet’s post hoc test (* *p* < 0.05).

**Figure 12:**
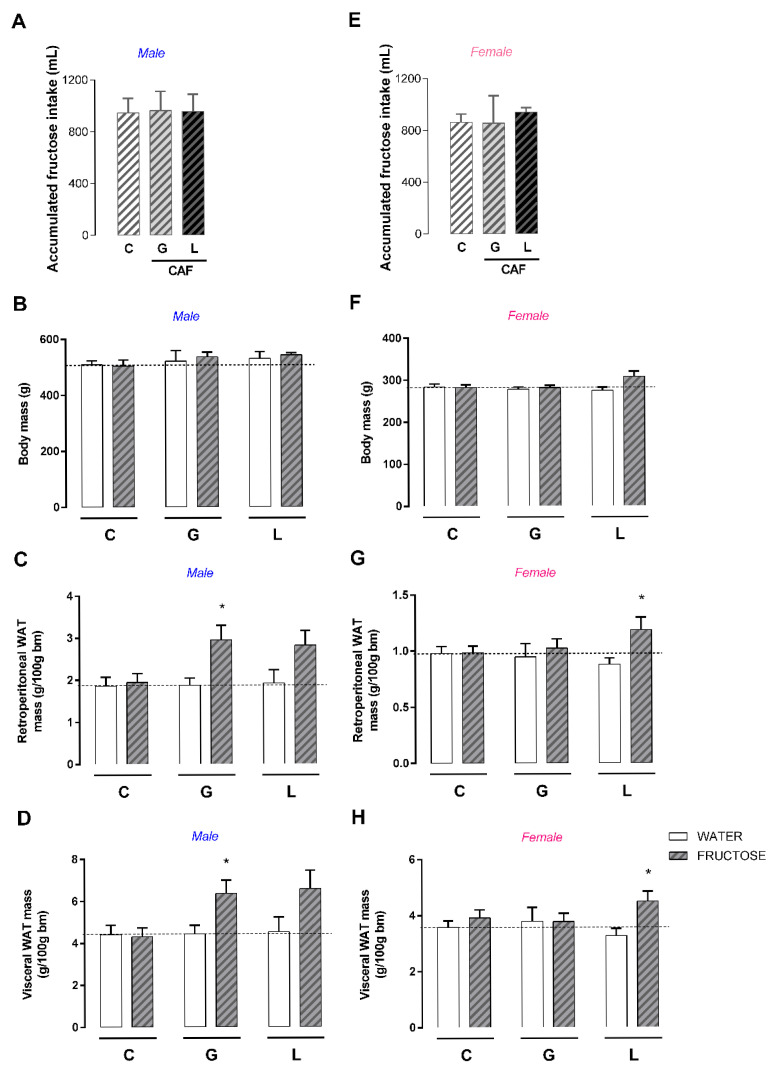
Effects of caffeine exposure during gestation (G) or lactation (L) period on metabolic response to fructose overload in adult male and female offspring. (**A**,**E**) Accumulated fructose intake; (**B**,**F**) body mass; (**C**,**G**) retroperitoneal WAT mass; (**D**,**H**) visceral WAT mass; WAT—white adipose tissue. Data are expressed as means ± SEM; *n* = 5–6. Statistical analysis was performed comparing the group water with the group fructose in each condition using multiple t test corrected by the Holm–Sidak method (* *p* < 0.05).

We evaluated hedonic food behavior and observed that CAF L male showed higher preference for an HFD (+12%; *p* < 0.05) (Figure 11A), whereas CAF G males showed a higher preference for sucrose (+22%; *p* < 0.05) (Figure 11B). Only CAF L female offspring showed a slightly lower preference for sucrose (4%; *p* < 0.05). Considering these hedonic behavior changes and energy imbalance with respect to intake, we subjected these adult offspring to a second metabolic insult: fructose overload. Although fructose intake did not change body mass, it promoted changes in adiposity according to the experimental group. Regardless of offspring sex, there were no differences in fructose intake between the experimental groups (Figure 12A,E), such as in body mass (Figure 12B,F). Among the male offspring, only the CAF G group—which consumed fructose—presented significantly greater retroperitoneal fat mass (+57%, *p* < 0.05) (Figure 12C) and visceral WAT (+43%, *p* < 0.05) (Figure 12D) mass than its respective control—lacked fructose. Among the female offspring, only the CAF L group exposed to fructose had significantly greater retroperitoneal (+35%; *p* < 0.05) (Figure 12G) and visceral WAT masses (+37%; *p* < 0.05) (Figure 12H) than the respective control.

## 4. Discussion

In this study, using an experimental model, we demonstrated that in addition to pregnancy, lactation is also a critical period for caffeine exposure, especially for female offspring. Maternal caffeine exposure at a low dose, corresponding to 250 mg/day in humans, restricted to the lactation period promoted glucose intolerance and increased susceptibility to obesity in response to fructose overload in female adult offspring. On the other hand, male offspring exposed to caffeine during the gestation period showed a greater preference for sugar and a greater susceptibility to obesity in response to fructose overload. Therefore, our data highlight sex-dependent effects according to the perinatal window of caffeine exposure. However, maternal caffeine exposure also promoted effects regardless of the window of exposure, such as lower T3 in weaned offspring.

In human and animal models, caffeine during the lactation period can affect iron levels and breastmilk volume [27,45,46,47], as reflected by the hematocrit of the baby [46]. It has also been reported that maternal caffeine exposure from birth until PND10 increases the locomotor activity [48], brain mass [49], and body mass of offspring [50]. Although these effects of caffeine during the lactation period have been reported, for the first time, we compared the different effects of maternal caffeine exposure during the gestation or lactation period on offspring health. Another goal of our work is the use of a safe dose according to the WHO, usually reached by the maternal diet during gestation [15,23] or lactation period [17]. The dosage of 25 mg/kg/day of caffeine in our experimental model is equivalent to 250 mg/day caffeine intake in humans [34], which corresponds to approximately two cups of coffee per day [35].

Even at this safe dose, caffeine intake during the continuous perinatal period, including during gestation and lactation, can promote reduced maternal body mass and adiposity, which can contribute to lower birth weight of female offspring [29]. Interestingly, here, we demonstrated that maternal caffeine intake during the gestation or lactation period did not impact maternal body mass or adiposity. However, the lower birth weight of the female offspring from dams exposed during gestation was maintained, suggesting the involvement of other factors. Some authors using a model of caffeine excess suggest a role for maternal corticosterone excess [51], which we did not observe in our model of low-dose caffeine. It is possible that the ability of caffeine to reduce placental blood flow can impair fetal nutrient apport [52], contributing to the lower birth weight of female offspring. Indeed, gene regulation in the placenta has been reported to differ according to fetal sex [53,54,55], which could explain the sexual dimorphism at birth. Interestingly, despite the strong correlation between low birth weight and increased risks of obesity and metabolic disorders throughout life [3], compared with CAF L female offspring and CAF G and L male offspring, CAF G female offspring were more protected from metabolic disturbances.

At the end of the lactation period, the corticosterone, insulin, and leptin levels of the dams exposed to caffeine, regardless of the window of caffeine exposure, were unchanged. However, they had lower T3 levels, which could be due to lower TSH activity in the gland. Although the TSH levels did not change, the expression of the TSH receptor in the gland decreased, which could impact its function even in the absence of changes in gland morphology. Although excess caffeine (150 and 200 mg/kg/day) during gestation promotes maternal hyperthyroidism [56], we previously demonstrated maternal hypothyroidism in dams exposed to low caffeine intake (25 mg/kg/day) during a continuous period of gestation and lactation [29]. Indeed, it is possible that caffeine modulates the intracellular content of cAMP [57] and affects the activity of TSH in thyroid hormone synthesis [58]. In dams, reduced T3 could contribute to lower brown adipose tissue mass and higher plasma cholesterol levels, affecting offspring indirectly through changes in milk composition. Here, we did not observe changes in the milk composition or hormone content, except for a tendency toward higher cholesterol levels in the breastmilk from dams exposed to caffeine during the lactation period, which did not affect the offspring’s plasma cholesterol. We did not measure T3 in the breastmilk, but a reduced T3 content could contribute to reduced plasma T3 in offspring exposed to caffeine during the lactation period. In addition, adaptive changes in the thyroid gland of weaned offspring could also impact hormone synthesis. Male offspring from the CAF G group presented a greater epithelial area, and an increased epithelial/colloid ratio, suggesting a greater stimulation by TSH. Unfortunately, for technical reasons, we did not measure TSH levels. Regardless, this supposed stimulation was not sufficient to increase thyroid hormone levels. Thus, the involvement of peripheral differences in deiodinase activity between male and female offspring should be considered to explain the lower T3 in males and in CAF L females [59]. We also cannot rule out the possibility that transfer of caffeine to offspring through breastmilk occurs through interactions with TSH. Indeed, the levels of caffeine in the plasma and breastmilk of mothers are correlated, reflecting the caffeine content in the plasma and tissue caffeine [28]. However, owing to sample volume limitations, we did not measure caffeine or TSH levels in weaned offspring. Despite these thyroid function changes, the adult animals did not show significant changes in plasma thyroid hormone levels, even in the presence of morphological changes in the gland, suggesting the presence of adaptive mechanisms. Interestingly, in a model of caffeine exposure during the gestation and lactation periods, offspring from both sexes presented higher T3 levels in adulthood [29].

Maternal caffeine exposure during gestation can affect glucose homeostasis, and conflicting findings have been reported, such as reduced [60,61] or increased insulin sensitivity [62] but increased beta cell apoptosis [63]. These experimental differences seem to be dependent on the caffeine dose. Here, we report unchanged glucose tolerance in offspring exposed to caffeine during the gestation period. Pancreas and adipose tissue also develop during the neonatal period [30], and it is plausible to find a different phenotype in offspring exposed to caffeine only during the lactation period. Indeed, we observed reduced glucose tolerance and normal insulin levels, suggesting lower insulin sensitivity, only in female offspring exposed to caffeine during the lactation period. This phenotype was not observed in a model of exposure to caffeine during the gestation and lactation periods [29]. Indeed, in vitro, caffeine directly reduces muscle insulin pathway activation [64], which in turn reduces insulin-stimulated glucose uptake [65].

Despite the normal body mass gain in adulthood, male offspring exposed to caffeine during gestation and female offspring exposed to caffeine during lactation exhibited lower cumulative food intake. This profile suggests lower energy expenditure, in accordance with the higher feed efficiency, which could favor the development of obesity in response to caloric overload. Despite the suggested lower energy expenditure, the animals did not exhibit changes in brown adipose tissue mass, adiposity, or thyroid hormone levels, except for a contradictory trend toward higher plasma T3 in female offspring from the CAF L group. In accordance with the hypothesis of increased susceptibility to obesity, male offspring from both groups showed a greater preference for a palatable diet. On the other hand, female offspring from the CAF L group showed a lower preference for sucrose solution.

Considering this metabolic profile and to test the hypothesis of increased susceptibility to obesity, we subjected the animals to a second metabolic insult, namely, exposure to fructose overload. Fructose, a sugar present in soft drinks, most often with caffeine, has potent lipogenic effects, promoting the development of obesity and other metabolic dysfunctions [66]. Indeed, in rats, compared with glucose or sucrose solution, fructose promoted higher triglyceride levels [67] and higher visceral adiposity and insulin resistance [68]. The harmful effects of fructose are a result of its potent lipogenic activity and ability to induce cellular stress, such as oxidative and endoplasmic reticulum stress, and inflammation [66], even in the absence of obesity [69]. In the liver, fructose is metabolized to generate energy without encountering rate-limiting and regulatory enzymes as glucose does. Therefore, increased pyruvate availability contributes to energy flux and de novo lipogenesis in the liver [70]. In fact, in rats and humans, excess fructose elevated postprandial triglyceride levels, increasing the VLDL fraction in plasma [71,72]. Excess quantities of these circulating lipids contribute to adipose tissue expansion, in addition to other fructose mechanisms, such as oxidative stress and its direct effect on the induction of adipogenesis, justifying their role in increasing adiposity [73]. Here, ten days of fructose overload alone did not increase the body mass or adiposity of control animals of either sex. However, the same fructose overload promoted greater adiposity in male offspring exposed to caffeine during the gestation period, such as in female offspring exposed to caffeine during the lactation period. Therefore, the susceptibility to obesity is increased in both sexes but in different windows of exposure. This adaptive response could involve higher expression of lipogenic factors such as sterol regulatory element-binding protein 1 (SREBP-1). Indeed, excess maternal caffeine (120 mg/kg day) during gestation increased SREBP-1 and fatty acid synthase (FAS) levels in the fetal liver [4]. Interestingly, the differences in the adiposity among experimental groups only appear after fructose overload, without changes in body mass, suggesting a transient effect. One limitation of our study is that we did not identify the molecular mechanisms involved in the offspring’s phenotype.

This susceptibility to metabolic dysfunction in early caffeine-exposed animals has been reported in response to a chronic postnatal HFD [4]. Despite the unchanged body mass, offspring from dams exposed to caffeine during gestation (120 mg/kg/day) and fed an HFD throughout life presented higher serum glucose and triglyceride levels than control animals in HFD of both sexes. In addition, animals exhibit increased hepatic lipid deposition and hepatic expression of lipogenesis and gluconeogenesis genes [4].

This increased susceptibility to obesity caused by early caffeine exposure is a concern; once, approximately 70% of American mothers consumed caffeine during the gestation period [14], but they reported reducing their caffeine intake [74,75]. However, some women restrict caffeine intake only during the gestation period and withdraw this restriction after baby birth. Therefore, excessive caffeine intake has been reported during the lactation period compared with the gestation period [16,17]. Thus, our model mimics a common finding in women during the perinatal period, highlighting the importance of amplifying and transmitting this caffeine restriction to the lactation period. Indeed, some organizations recommend total caffeine withdrawal during the gestation and lactation periods [76]. However, the WHO and others did not devote attention to lactation [24,77]. Our work revealed the importance of this critical period to maternal caffeine intake and its potential contribution to obesity development in an experimental model. In addition, fructose overload is common in the adult population because of the elevated intake of soft drinks and other ultra-processed foods [31]. Thus, our experimental model is an interesting tool for investigating the different effects of a maternal caffeine diet during the perinatal period, which mimics a common human condition.

Although the caffeine metabolism in rats and humans are comparable, some differences exist [78,79], and careful extrapolation of our findings to humans is important. Other limitations of our experimental model are the duration of the gestation and lactation periods between species and fructose metabolism. However, many works support the deleterious impact of maternal caffeine intake in both species [12,79]. Therefore, here, we highlight the deleterious impact of caffeine exposure during the lactation period, even at a safe dose, in a rodent experimental model. These findings highlight the importance of caffeine intake for breastfeeding women.

## 5. Conclusions

Maternal caffeine intake during gestation or lactation may differentially affect offspring metabolism and thyroid function. Despite lower T3 at weaning, these changes are not observed in adulthood. Other hormones, such as insulin, leptin, and corticosterone, are not affected. Exposure only during lactation promoted glucose intolerance and increased susceptibility to obesity in response to fructose in female offspring. On the other hand, exposure only during gestation promoted a greater preference for a palatable diet and increased susceptibility to obesity in response to fructose in male offspring. The results of the current model system suggests that the effects of caffeine on offspring health depend on the window of caffeine exposure and the sex of the offspring. Therefore, for female offspring, the lactation period seems to be a development window that is more critical to early caffeine effects. The mechanisms involved in this offspring phenotype, such as in the response to fructose overload, should be clarified in the future.

## Figures and Tables

**Table 1:** Diet composition of chow used in palatable test.

Composition	STD	HFD
Carbohydrates (%)	63	44
Protein (%)	26	14
Lipids (%)	11	42
Energy (Kcal/g)	3.36	4.71

**Table 2:** Primer’s sequence of evaluated genes.

Gene	Forward Sequence	Reverse Sequence
Thyroid stimulating hormone receptor (*Tshr*)	GTACTTCTCCACCCTGCGAA	GCTCGAAAAGGCAAGACTGG
Hypoxanthine phosphoribosyltransferase 1 (*Hprt*)	GCAGTACAGCCCCAAAATGG	AACAAAGTCTGGCCTGTATCCAA
Ribosomal protein lateral stalk subunit P0 (*Rplp0*)	TTCCCACTGGCTGAAAAGGT	CGCAGCCGCAAATGC

**Table 3:** Breastmilk composition from dams exposed to caffeine during gestation or lactation period.

Breastmilk Levels	Control	CAF G	CAF L
Lactose (mg/mL)	21.9 ± 3.3	18.5 ± 4.8	22.9 ± 3.1
Protein (mg/mL)	11,452 ± 520	11,280 ± 491	11,338 ± 518
Triglycerides (mg/dL)	3927 ± 249	4354 ± 267	4357 ± 277
Energy (Kcal/100 mL)	92.7 ± 3.3	95.1 ± 4.7	96.5 ± 3.3
Insulin (ng/mL)	7.31 ± 1.57	6.23 ± 1.51	7.02 ± 0.66
Leptin (ng/mL)	7.00 ± 1.22	6.52 ± 0.72	6.59 ± 0.79

CAF G—caffeine exposure during gestation period; CAF L—caffeine exposure during lactation period; data are expressed as means ± SEM; *n* = 4–5. Statistical analysis was performed using one-way ANOVA followed by Dunnet’s post hoc test.

**Table 4:** Plasma hormones of dams and offspring at weaning.

Hormone	Control	CAF G	CAF L
DAMS			
Insulin (ng/mL)	0.79 ± 0.11	0.65 ± 0.05	0.83 ± 0.10
Leptin (ng/mL)	1.44 ± 0.31	1.35 ± 0.34	1.47 ± 0.29
Corticosterone (pg/mL)	96,040 ± 27,180	86,780 ± 14,070	79,460 ± 16,490
MALE OFFSPRING (PND21)			
Insulin (ng/mL)	0.09 ± 0.01	0.14 ± 0.03	0.12 ± 0.02
Leptin (ng/mL)	0.60 ± 0.13	0.40 ± 0.05	0.43 ± 0.09
Corticosterone (pg/mL)	191,610 ± 35,220	157,270 ± 18,910	157,190 ± 31,340
FEMALE OFFSPRING (PND21)			
Insulin (ng/mL)	0.11 ± 0.02	0.10 ± 0.01	0.12 ± 0.02
Leptin (ng/mL)	0.41 ± 0.11	0.23 ± 0.09	0.45 ± 0.11
Corticosterone (pg/mL)	182,820 ± 16,280	147,120 ± 26,130	176,480 ± 44,790

CAF G—caffeine exposure during gestation period; CAF L—caffeine exposure during lactation period; data are expressed as means ± SEM; *n* = 5–6. Statistical analysis was performed using one-way ANOVA followed by Dunnet’s post hoc test.

**Table 5:** Plasma hormones of dams and offspring in adulthood.

Hormone	Control	CAF G	CAF L
MALE OFFSPRING (PND180)			
Insulin (ng/mL)	2.44 ± 0.47	2.36 ± 0.28	2.47 ± 0.41
Leptin (ng/mL)	1.83 ± 0.88	1.52 ± 0.26	4.19 ± 1.59
Corticosterone (pg/mL)	76,250 ± 76,250	102,410 ± 10,710	101,360 ± 12,320
FEMALE OFFSPRING (PND180)			
Insulin (ng/mL)	3.64 ± 0.44	4.02 ± 0.45	3.52 ± 0.30
Leptin (ng/mL)	0.90 ± 0.23	1.11 ± 0.29	0.48 ± 0.24
Corticosterone (pg/mL)	81,770 ± 9300	85,990 ± 9970	94,560 ± 8980

CAF G—caffeine exposure during gestation period; CAF L—caffeine exposure during lactation period; data are expressed as means ± SEM; *n* = 5–6. Statistical analysis was performed using one-way ANOVA followed by Dunnet’s post hoc test.

## Data Availability

The original contributions presented in this study are included in the article. Further inquiries can be directed to the corresponding author.
